# Supplementary material for: Evaluation of the practicability of a finger-stick whole-blood SARS-Cov-2 self-test adapted for the general population
Source: PLoS One. 2021 Jan 28;16(1):e0245848. doi: 10.1371/journal.pone.0245848 (PMC7842899; doi:10.1371/journal.pone.0245848)
Supplement: Attachment — Submitted filename: Reviews PONE rebuttal.docx [file pone.0245848.s002.docx]

PONE-D-20-21033
Evaluation of the Practicability of a Finger-Stick Whole-Blood SARS-Cov-2 Self-Test Adapted for the General Population.

Dear PLoS one editors and reviewers,

The authors would like to thank you all for your time and expertise you brought into this review. Please find below our responses (in blue) to every of your remarks and editing requests.

We hope that, following this revision, you will find that this manuscript reaches the scientific standards of publication in PLoSOne.

*PLOS ONE*

*1. Please ensure that your manuscript meets PLOS ONE's style requirements, including those for file naming. The PLOS ONE style templates can be found at*

[*https://journals.plos.org/plosone/s/file?id=wjVg/PLOSOne_formatting_sample_main_body.pdf*](https://journals.plos.org/plosone/s/file?id=wjVg/PLOSOne_formatting_sample_main_body.pdf) *and*

[*https://journals.plos.org/plosone/s/file?id=ba62/PLOSOne_formatting_sample_title_authors_affiliations.pdf*](https://journals.plos.org/plosone/s/file?id=ba62/PLOSOne_formatting_sample_title_authors_affiliations.pdf)

We have amended the manuscript following this editorial guidelines

*2. Please provide additional details regarding participant consent. In the ethics statement in the Methods and online submission information, please ensure that you have specified (1) whether consent was informed and (2) how oral consent was documented and witnessed. If your study included minors, state whether you obtained consent from parents or guardians.*

A sentence was added (line 150) to specify how consent was obtained. This study did not include minors.

*3. In your methods section, please provide the names of the four different locations volunteers were selected from.*

The names of the nuclear plants were added (line 157), the other two (Vaccination clinic of the CHR Orleans and The CHR Orleans) were already mentioned.

*4. Please discuss whether participants were able to opt out of the study and whether individuals who did not participate received the same treatment offered to participants.*

Most of the patients were healthy volunteers as this study is not about treatment. However, when applicable, participants were treated according to the standard of care.

*5.We note that you have indicated that data from this study are available upon request. PLOS only allows data to be available upon request if there are legal or ethical restrictions on sharing data publicly. For information on unacceptable data access restrictions, please see* [*http://journals.plos.org/plosone/s/data-availability#loc-unacceptable-data-access-restrictions*](http://journals.plos.org/plosone/s/data-availability#loc-unacceptable-data-access-restrictions)*.*

The Database is made available as a supplementary information file

*6. We note you have included a table to which you do not refer in the text of your manuscript. Please ensure that you refer to Table 1 in your text; if accepted, production will need this reference to link the reader to the Table.*

Reference to Table 1 was added line 168

**Reviewers' comments:**

**Comments to the Author**

*Reviewer #1:*

*The manuscript “Evaluation of the Practicability of a Finger-Stick Whole-Blood SARS-CoV-2 Self-Test Adapted for the General Population” describes the results of a usability study conducted to evaluate the suitability of use of the COVID-PRESTO rapid test by the general population. The COVID-PRESTO test is a lateral flow chromatographic immunoassay that has been developed to detect and differentiate IgG and IgM antibodies to SARS-CoV-2 in human fingerstick whole blood. The study aims to assess the practicability of the test when used in non-laboratory settings. The study included 142 participants with no experience in blood drawing, whose capability to obtain an interpretable result and ability to read the results were evaluated. 88.4 % of the users judged the instructions for use to be clear and understandable. 99.3 % of the users obtained a valid result and, according to the supervisors, 92.7% of the tests were properly performed by the user. The results suggest that the test shows a potential to be used as a self-test.*

*Major comments:1. The authors refer to the test used in the study as a “rapid diagnostic test”. However, generally, a diagnostic test is a test that detects an acute infection, i.e. an RT-PCR or an antigen test. Antibody assays identify individuals with an adaptive immune response, which indicates prior rather than acute infection.*

Agreed, we changed the nature of test to “Point of Care test, POC” throughout the document. This is consistent with the published performance paper as well.

*2. Lines 230 – 233: it is stated that “Around a hundred of COVID-19 serological tests are currently commercially available in the US through the Emergency Use Authorization (EUA) granted to the US Centers for Disease Control and Prevention by the Food and Drug Administration (FDA)”. This statement is not correct. In fact, an EUA is granted by FDA to every manufacturer of a test, not to CDC, and as of 08/09/2020, there are 35 FDA-authorized serological tests (*[*https://www.fda.gov/medical-devices/coronavirus-disease-2019-covid-19-emergency-use-authorizations-medical-devices/vitro-diagnostics-euas#individual-serological*](https://www.fda.gov/medical-devices/coronavirus-disease-2019-covid-19-emergency-use-authorizations-medical-devices/vitro-diagnostics-euas#individual-serological)*).*

This reference has been cross checked and we have modified this statement in the text to reflect the reviewer’s comment in (lines 83 – 100)

*3. In Discussion (lines 275 – 276), a conclusion is made that the COVID-PRESTO is judged practical with a global satisfaction rate of 95% by the users and is favorably seen as a potential self-test. I believe that users’ satisfaction rate is not an indicator of a test’s suitability to be used outside professional healthcare facilities. The practicability of an assay as a self-test should be determined by the ability of the end user to correctly perform the test, as evaluated by a trained professional. A satisfaction questionnaire can be used to evaluate the ease of understanding the instructions for use.*

This point has been added in the discussion (lines 343 – 350). The ability of the user to correctly interpret the results has been tested in our study (sub-study 2) and the outcome was clearly positive (99% of correct answers).

*4. The main conclusion that the authors make is that the COVID-PRESTO assay “should be considered as a suitable candidate for a public release”. I think it should also be noted that improvements need to be made to the test’s instructions for use (or other mitigating measures need to be put in place) because 24.6% of participants asked for assistance when administering the test.*

This point is discussed in the paper (lines 366 – 375), and we acknowledge that changes need to be made in the instruction materials to increase their comprehension. The instruction leaflet and video will be amended to address the points that were considered not clear by the users.

*5. In addition, although the study results seem to suggest that the test may be suitable as a self-test for the general population, the authors have to keep in mind that there may be different regulatory requirements in different parts of the world that need to be met in order for the test to be approved for this specific use.*

The conclusion of the paper is that COVID-PRESTO should be considered as a **candidate** for self-testing. Currently, this test is intended for the French market only. The French competent authority considers that it is premature to promote the use of SARs CoV-2 self-test due to a lack or published data regarding the reliability of these tests in real life conditions. Cf below (document in French):

https://www.has-sante.fr/upload/docs/application/pdf/2020-05/rapport_tests_serologiques_rapides_covid-19_vd.pdf

We hope that the results presented here will help fill that void and fuel the discussion about these tests.

*Minor comments: Consulting a professional editor will improve the manuscript’s readability by minimizing grammatical inaccuracies.*

The manuscript has been reviewed by a native English speaker in order to correct the grammatical inaccuracies

*Reviewer #2: SUMMARY*

*In this study, the authors evaluate the practicability of the AAZ COVID-PRESTO® test, a Rapid Diagnostic Test (RDT), easy-to-use device to detect specific antibodies to SARS-CoV-2 in human whole blood.*

*In late 2019 the emergence of SARS-CoV-2, which causes COVID-19, initiated a pandemic that has been associated with over 500,000 deaths globally as of July 2020. The original outbreak that started in Wuhan, China, rapidly expanded into other continents.*

*AAZ COVID-PRESTO® is a lateral flow immunochromatographic assay that can be conducted using a single drop of blood. The test uses anti-human IgM antibody (test line IgM), anti-human IgG antibody (test line IgG) and rabbit IgG (control line C) immobilized on a nitrocellulose strip. The Conjugate (recombinant COVID-19 antigens labeled with colloidal gold) is also integrated into the strip.*

*When a specimen is added to the sample well (S) followed by assay buffer added to the buffer well (B), IgM &/or IgG antibodies if present, will bind to COVID-19 conjugates forming immunocomplexes. These complexes migrate through the nitrocellulose membrane by capillary action. When complexes meet the line of the corresponding immobilized antibody (anti-human IgM &/or anti-human IgG), they are captured forming a burgundy colored band which confirm a reactive test result. Absence of a colored band in the test region indicates a non-reactive test result. If the proper volume of specimen has been added, a colored line will change from blue to red in the control line region.*

*The authors describe a study conducted in 142 volunteers selected from four different locations in Central France from March 20th through May 5th, 2020. Each volunteer had to perform the following tasks:(i) read test’s instructions or watch instructional video, (ii) use the lancet to prick the fingertip and extract a drop of blood, (iii) conduct the test, (iv) read and interpret test results and (v) fill out a questionnaire.*

*Finally, the authors concluded that the COVID-PRESTO® assay was successfully used by most participants and showed a promising potential as a self-test to be used by the general population.*

*MERITS*

*In late 2019 the emergence of SARS-CoV-2, which causes COVID-19, initiated a pandemic that has been associated with over 500,000 deaths globally as of July 2020. The original outbreak that started in Wuhan, China, rapidly expanded into other continents. In United States, novel coronavirus has spread to nearly every state and territory, accounting for at least 192,000 deaths since February 2020.*

*The topic of this manuscript is extremely relevant in the current stage of the pandemic.*

*Containment strategies have focused on travel restrictions, isolation, use of masks, and contact tracing. As governments start easing these rules, and citizens return to daily but restricted activities, health and research authorities are searching for effective ways to test, control and track every case in large populations.*

*In this context, the development of new easy-to-use tests that could be accessible and distributed to everyone is imperative to help control the spread of SARS-CoV-2. After a week from the first clinical manifestations, the sensitivity of RT-PCR tests diminishes gradually, due to the decreasing amount of virus particles in the respiratory tract epithelium. In such cases, patients may have false negative results, despite the ongoing infection. IgG and IgM antibodies against the virus can be detected with 1-3 weeks after exposure.*

*Serological self-administered tests are of great interest, to assess the degree of immunization, to detect asymptomatic cases, to trace contacts, and to support decisions to re-admit people at work, schools and universities. Indeed, these tests could become crucial in supporting public health efforts to track virus spread and to develop containment strategies.*

*This work has several strengths:*

*• It provides additional studies of the suitability of the COVID-PRESTO® test needed to show its performance in untrained individuals as a self-administered assay. These studies are required by the European Centre for Disease Prevention and Control, before a test can be made available to the public.*

*• The assay was tested in non-medical volunteers, to avoid bias due to (i) previous or current experience in blood drawing and (ii) familiarity with interpretation of diagnostic tests.*

*• The test’s instructions can be followed in two different formats (printed or video), which expands accessibility to materials.*

*• The results showed that the execution of the test was easily conducted by a wide range of individuals independently of age or education level without the need of previous training.*

*• This rapid test has the potential to be used for screening in low resource or emergency settings, such as evacuation or refugee centers.*

*CRITIQUE*

1. *Introduction*
a. *This section provides a generalized background of the topic. The authors may wish to provide a brief description of the AAZ COVID-PRESTO®, how it’s executed and indicate some of its characteristics, including specificity and sensitivity.*

Added: lines 114 to 121

b. *The paper that you reference on Page 3, line 92, has been published. The sentence starting “It has been evaluated …” (Line 90) will need to be edited to reflect that.*

Corrected: lines 118 to 121

c. *Interpretation of Figure 1 will benefit of a caption with a brief explanation. In addition, please define all abbreviations used.*

An explanation has been added in lines 130 to 143

d. *Minor edits*

*Please correct the following sentences:*

*(d1) Line 75: add a coma between resource center and the COVID-19 pandemics.*

Corrected

*(d2) Line 77: add a stop after health emergency and start the next sentence as “The mark of…”*

Corrected

*(d3) Line 81: change the sentence from “In most countries these measures have been effective in slowing down the transmission of the virus” to “In most countries these measures have been effective in slowing down virus transmission”.*

Corrected

*(d4) Line 96: change the sentence from “The aim of this study was to evaluate the COVID-PRESTO® test in terms of participants’ capability to obtain an interpretable result, and the users’ ability to interpret the results” to “The aim of this study was to evaluate the COVID-PRESTO® assay in terms of participants’ capability to obtain a valid test, and the users’ ability to interpret the results”.*

Done

2. *Methods*
e. *The first sentence of the Data Analysis section needs clarification. It’s not possible to understand how the authors described the statistical analysis of the populations.*

We rephrased this section in order to clarify our methods.

*f. Minor edits*

*Please correct the following:*

*(f1) Table 1 needs formatting. Please move the title to the empty row and add a section header (Summary and explanation of the test) so that it aligns with the format you used in subsequent sections of your table.*

Corrected

*(f2) Section 1, Table 1: capitalize the first word of each sentence.*

Corrected

*(f3) Sub-study 2 section: the last two sentences need editing. Please consider changing them to “The participant had to randomly choose three tests and write down the results. A supervisor was in charge collecting and assessing their responses”.*

Corrected

*(f4) Table 2, question 3: change to “Did you manage to collect the recommended amount of blood?”*

Corrected

*(f5) Table 2, question 6: change to “Did you obtain a valid result (at least one band)?”*

Corrected

*(f6) Table 3: please change the title to “Supervisor’s Feedback”.*

Corrected

3. *Results*

*The results overall support the finding that the COVID-PRESTO® assay is an easy-to-use test and has promising potential to be used by the general population.*

*g. Table 4*

*(g1) Please explain what’s the difference between N(%) and % (under characteristics) and add a legend to the table.*

It was a mistake, there was no difference, the table has been corrected accordingly.

*(g2) In Sub-study 1, you indicate that N=142. However, only in the age category you have 142 volunteers while under study site, age and education level you have a sample of 140. Please explain the reason for this discrepancy and make changes to the totals if needed.*

The entire table has been corrected. Errors were spotted after initial submission of the manuscript to PLoSOne. We addressed them in the revised manuscript. Proportions changed a little but nothing significant was observed.

*(g3) Sub-studies 1 and 2: Although the results are explained in the next, it is difficult to follow the outcomes related to the different groups and tasks you tested. Bar graphs, pie charts or additional tables could be helpful to better illustrate the findings and help the reader follow the analysis.*

Bar graphs and a pie chart were added in the results section to increase clarity.

*h. Minor edits*

*Please correct the following:*

*(h1) Line 147: change “to Sub-study 2” to “in Sub-study 2”*

Corrected

*(h2) Lines 148 and 149: change “classes” to categories”.*

Corrected

*(h3) Line 149: the sentence should read “... level categories were defined according to the International Classification of Education …”.*

Corrected

*(h4) Line 195: change “assistance to the supervisor” to “supervisor’s assistance”.*

Corrected

*(h5) Line 216: change “…any influence of the ability of the participant…” to “…any influence in the ability of the participant…*

Corrected

4. *Discussion*

*Findings are discussed in the context of the published literature and the references cited are relevant to the study. The data supports the conclusions of the work.
The discussion is long. The paragraphs related to the impact that the serology tests can have in fighting the current epidemic, regulations imposed by FDA, CDC and European health authorities and the need to assess the practicability of self-administered assays before promoting their use, belongs to the introduction of the paper.*

The entire paragraph and the following has been moved to the introduction (lines 83 – 113)

*g. Minor edits*

*(g1) Line 262: delete “These results are currently…” and add the reference after the previous sentence.*

Corrected
