# Supplementary material for: Evaluation of the practicability of a finger-stick whole-blood SARS-Cov-2 self-test adapted for the general population
Source: PLoS One. 2021 Jan 28;16(1):e0245848. doi: 10.1371/journal.pone.0245848 (PMC7842899; doi:10.1371/journal.pone.0245848)
Supplement: Attachment — Submitted filename: Reviews PONE rebuttal 2nd revision.docx [file pone.0245848.s003.docx]

PONE-D-20-21033
Evaluation of the Practicability of a Finger-Stick Whole-Blood SARS-Cov-2 Self-Test Adapted for the General Population.

Dear PLoS one editors and reviewers,

The authors would like to thank you all for your time and expertise you once again brought into this review. Please find below our responses (in blue) to every of your remarks and editing requests.

We sincerely apologize for the fact that some of the previously requested changes did not make it into the submitted revised manuscript. We are confident that we have now addressed all the reviewers’ comments.

We hope that, following this revision, you will find that this manuscript reaches the scientific standards of publication in PLoSOne.

*Reviewer #1: Authors state that the revised version of the manuscript was proofread by a native English speaker, but the paper still contains multiple grammatical errors and missing/extra words.*

The manuscript was edited by a native English speaker once again and the certificate is included with this re-submission

*I suggest authors check the references: after several paragraphs were moved from discussion to introduction, the references are out of order. In addition, the reference to the FIND foundation website is not formatted as a reference (line 79).*

The references have been fixed.

**Methods and Materials:**

*Substudy 1 (lines 157 – 158): It is not clear from the sentence whether the volunteers read the test’s Instructions for Use, watched an instructional video, or both.*

The sentence was edited into: “Two different instructional media were available: written Instructions for Use and an instructional video. Each volunteer was invited to use either one or both, in order to get a clear understanding of how to perform the test.”

**Results:** *Authors indicate that 142 volunteers were selected to participate, and 140 of those filled in the satisfaction questionnaire. All sections of Table 4 therefore include 140 participants, with the exception of the Age section, which lists 142 participants, and the N in the Substudy 1 table heading. Please revise the table so that the numbers are consistent.*

*Substudy 1 (lines 223 and 232): the number of participants who filled in the questionnaire is 141 in this section of the text, which contradicts the previous paragraphs and Table 4, and it appears that this number was used in all percentage calculations in these two paragraphs. Please revise the numbers so that the number of participants who filled in the questionnaire is consistent throughout the manuscript.
Substudy 2 (line 260): the description of the study specifies that the number of participants who filled in the questionnaire is 141. Please revise the numbers so that the number of participants who filled in the questionnaire is consistent throughout the manuscript.*

The numbers have been re-checked and the table in the main text as well as the dataset have been corrected

***Discussion*** *(lines 303 – 306): The order of these two sentences seems to suggest that the first sentence (“Nevertheless, we observed…”) supports the conclusion in the second sentence (“This strongly suggests that the execution...”). Since it doesn’t appear that the authors intended for these two sentences to be logically linked, I suggest revising the passage or at least changing the order of the sentences.*

The order of the sentences has been changed

*Although seropositivity typically occurs later in the course of disease, as authors correctly noted, based on the currently available data it is not clear whether the antibody positivity confers immunity. In addition, infection status cannot be inferred from a result of a serological test. To this end, the COVID-PRESTO test IFU will recommend further molecular testing to diagnose acute infection; however, this instruction seems to only apply in case of a positive IgM test. It has been shown that for SARS-CoV-2 IgM and IgG seroconversion times are not significantly different, and some studies reported that up to 60% of patients were positive for IgG within the first seven days since onset of symptoms (PMID: 32964627). Therefore, I would suggest not excluding IgG positive patients from this recommendation in cases where acute infection is suspected.*

We agree, the distinction between IgM/and IgG has been removed by deleting IgM, as requested

Reviewer #2: MERITS

The manuscript has improved substantially after the authors edited the text, added graphs and addressed reviewers’ comments. However, there are still several changes/edits that need to be included. I mentioned several examples (see minor edits), but I strongly encourage the authors to consult a scientific editor.

MAJOR ISSUES

*1. Table 4: Although the authors explained that there is no difference between N(%) and % (under characteristics) and that they state that the table was updated, Table 4 as it stands in the revision, looks identical to the one submitted in the original manuscript. In addition, no changes to the totals or proportions were made. Please update Table 4 accordingly.*

Table 4 has been updated

2. *The comparisons conducted to determine whether there was a difference in participants’ comprehension when instructions were delivered in writing or by video, are not clear. Addition of a table (including groups and results) may facilitate readers’ understanding.*

The data have been summarized in a new Table 5

*3. Figure 3: what does the asterisk over the column indicate? Please add this information in the figure legend.*

The asterisk indicates that a p value < 0.05 was calculated using a Chi2 test vs the other education levels. The information has been added to the figure legend

MINOR EDITS

*4. Line 117: delete easy-to-use device*

Deleted

*5. Line 118: replace “In summary” by “Briefly”*

Replaced

*6. Line 136: delete “when” and capitalize “A”.*

Done

*7. Line 137: replace “followed by assay buffer added to the buffer well” with “followed by addition of assay buffer to the buffer well”.*

Replaced

*8. Line 138: delete the “s” in “migrates” (complexes is plural).*

Done

*9. Line 155: Add a stop after “(if any)”.*

Done

*10. Line 158: delete “the” before study. Start sentence with “Study volunteers…”*

Deleted

*11. Lines 168-169: the sentence needs further editing for clarity. Replace “information” by “instructions”.*

Done

*12. Line 170: sentence should read “The instructions WERE as follows…”*

Modified

*13. Line 177: delete “on the test”.*

Rephrased

*14. Line 184: change title to “Reading and interpretation of results”.*

Changed

*15. Line 188: replace “their correctness” to “accuracy”.*

Replaced

*16. Please revise reference # 2. The citation is incomplete.*

The reference has been updated

*17. Table 2, question 3. I suggested the authors to change this question to “Did you manage to collect the recommended amount of blood?” In their response, they state that they made the requested modification. However, Table 2 as it stands in the revision, does not reflect the change.*

Modified

*18. Table 2, question 6. I suggested the authors to change this question to “Did you obtain a valid result (at least one band)?” In their response, they state that they made the requested modification. However, Table 2 as it stands in the revision, does not reflect the change.*

Modified

*19. Table 3. I suggested the authors to change the title and table heading to “Supervisor’s Feedback”. In their response, they state that they made the requested modification. However, Table 2 as it stands in the revision, does not reflect the change.*

Done

*20. Line 199: delete the word “their”.*

Deleted

*21. Line 205: add a comma after “among those”.*

Added

*22. Line 208: add “:” after defined (…categories were defined:…).*

Added

*23. Line 211: number “6” is missing.*

Added

*24. Line 220: replace “on the 221 comprehension of the instructions notice” with “ on comprehension’s instruction”.*

Replaced by “instructions’ comprehension”

*25. Line 222: add a comma after “However”.*

Added

*26. Line 223: add a comma after “Indeed”.*

Added

*27. Line 258: delete the word “classes” after age and education.*

Done

*28. Line 273: same as in line 258.*

Done

7. PLOS authors have the option to publish the peer review history of their article ([what does this mean?](https://journals.plos.org/plosone/s/editorial-and-peer-review-process#loc-peer-review-history)). If published, this will include your full peer review and any attached files.

If you choose “no”, your identity will remain anonymous but your review may still be made public

**Do you want your identity to be public for this peer review?** For information about this choice, including consent withdrawal, please see our [Privacy Policy](https://www.plos.org/privacy-policy).

Reviewer #1: No

Reviewer #2: No
